# Beyond Technological Access: Exploring Contextual Factors Related to Teleassessment Usability in Interstitial Lung Disease

**DOI:** 10.3390/ijerph23080944

**Published:** 2026-07-23

**Authors:** Wallace Pereira Silva, Giovanna Camargo Mello, Deborah Madeu Pereira, Cid André Fidelis De Paula Gomes, Carla Malaguti, Luciana Maria Malosa Sampaio, Soraia Micaela Silva

**Affiliations:** 1 Postgraduate Program in Rehabilitation Sciences, Universidade Nove de Julho (UNINOVE), São Paulo 01504-001, SP, Brazil; wallace.ps97@hotmail.com (W.P.S.); giovannacamargomello98@gmail.com (G.C.M.); cid.andre@gmail.com (C.A.F.D.P.G.); lucianamalosa@gmail.com (L.M.M.S.); 2 Department of Pneumology, Santa Casa de Misericórdia Hospital, São Paulo 01221-020, SP, Brazil; dra.deborahmadeu@gmail.com; 3 Postgraduate Program in Rehabilitation Sciences and Physical-Functional Performance, Federal University of Juiz de Fora (UFJF), Juiz de For a 36036-900, MG, Brazil; carla.malaguti@ufjf.br

**Keywords:** telehealth, interstitial lung disease, usability, TUQ, contextual factors

## Abstract

**Highlights:**

**Public health relevance—How does this work relate to a public health issue?**

- Teleassessment of individuals with interstitial lung disease (ILD) is feasible via video consultation but its usability is shaped by socioeconomic and contextual factors beyond device ownership.

**Public health significance—Why is this work of significance to public health?**

- Income, perceived clinical feasibility, and intensity of access barriers were significantly associated with teleassessment usability, highlighting digital inequity as a determinant of healthcare access.

**Public health implications—What are the key implications or messages for practitioners, policy makers and/or researchers in public health?**

- Equitable implementation of teleassessment in chronic respiratory care requires attention to digital literacy, socioeconomic conditions, and clear clinical criteria for appropriate use of remote assessment.

**Abstract:**

Telehealth has the potential to improve access to healthcare, but teleassessment usability may be influenced by contextual factors beyond technology access alone. This study investigated factors associated with teleassessment usability among individuals with interstitial lung disease (ILD) in Brazil using a convergent mixed methods design with 31 adults from four Brazilian regions. Usability was assessed with the Telehealth Usability Questionnaire (TUQ-Brazil); associations with contextual variables were examined using Spearman’s correlation. Qualitative data were obtained through semi-structured interviews and analyzed using thematic analysis. Participants generally reported acceptable usability perceptions with predominance of agreement responses; greater dispersion was observed for items related to equivalence with in-person care and video quality. Usability was significantly associated with income (rho = 0.437; *p* = 0.014), perceived clinical feasibility (rho = −0.397; *p* = 0.027), and perceived intensity of access barriers (rho = −0.428; *p* = 0.016). Qualitative findings identified financial constraints, digital literacy, internet stability, third-party support dependence, and absence of physical examination as key experiential factors. Teleassessment was perceived as a complement for follow-up and triage rather than a substitute for face-to-face assessment. Usability in ILD is shaped by socioeconomic, clinical, and structural factors, underscoring the need to address digital inequalities in telehealth implementation.

## 1. Introduction

Interstitial lung diseases (ILD) comprise a heterogeneous group of chronic respiratory conditions associated with substantial morbidity, mortality, and an increasing burden on health systems worldwide. According to the 2019 Global Burden of Disease (GBD) Study, the age-standardized global prevalence of ILD was 57.62 cases per 100,000 population. Between 1990 and 2019, the global burden of ILD increased substantially, with marked increases in incident cases, mortality, and years of life lost [1]. Despite this growing burden, epidemiological data from Latin America remain scarce. Among the GBD regions analyzed between 1990 and 2021, Andean Latin America showed one of the largest relative increases in the prevalence of ILD and pulmonary sarcoidosis [2]. In Brazil, a multicenter cohort including 1406 patients from six referral centers identified connective tissue disease-associated ILD as the most frequent etiology (27%), followed by hypersensitivity pneumonitis (23%), idiopathic pulmonary fibrosis (14%), unclassifiable ILD (10%), and sarcoidosis (6%) [3].

ILD progression frequently culminates in irreversible respiratory failure and premature death, which is reflected in the predominance of years of life lost (YLLs) within overall DALYs for this disease group. Beyond its clinical impact, the economic burden of ILD is substantial. A systematic review of cost-of-illness studies reported direct medical costs ranging from USD 1824 to USD 116,927 annually per patient and highlighted inpatient care, outpatient visits, and medication as the major contributors to these costs [4]. Indirect costs related to productivity loss and caregiver burden further compound this impact. Access to pulmonary rehabilitation and specialized multidisciplinary care remains particularly constrained in low- and middle-income countries, where structural limitations and socioeconomic inequalities restrict timely diagnosis and continuity of care.

In this context, telehealth has been proposed as a strategy to expand access to care by reducing geographic barriers and optimizing the use of specialized resources, particularly relevant for individuals with ILD, who often present with mobility limitations, dyspnea, and fatigue that compound the burden of travel to in-person appointments [5]. Evidence from a study conducted in South Africa highlighted that the absence of physical examination is consistently perceived by users as a structural weakness of remote care models, and that telehealth provision in certain contexts has been concentrated in the private sector, raising concerns about equity of access in low- and middle-income settings [6]. Conversely, studies conducted in Brazil have generally reported favorable feasibility, acceptability, and patient satisfaction across different telehealth initiatives, including primary care services, telehealth networks within the Brazilian Unified Health System (SUS), and videoconference-based teleassessment in individuals with chronic stroke [7,8,9].

Although positive user satisfaction is frequently reported across this literature [10], satisfaction alone does not fully capture the multidimensional construct of usability. Unlike satisfaction, usability encompasses multiple dimensions related to users’ interactions with health technologies, making it a broader construct for evaluating remote care modalities. Structured instruments capable of capturing this multidimensionality are therefore necessary to meaningfully evaluate remote care modalities [11]. The Telehealth Usability Questionnaire (TUQ) was developed for this purpose and assesses usability through a multidimensional construct encompassing usefulness, ease of use, effectiveness, reliability, and satisfaction, enabling a comprehensive assessment of user experience from the perspectives of both patients and healthcare professionals [11]. The instrument has since been translated and cross-culturally adapted into Brazilian Portuguese (TUQ-Brazil), preserving its original domain structure while demonstrating adequate psychometric properties for use in Brazilian Portuguese-speaking populations [12].

An important conceptual distinction should be made between telemonitoring, telerehabilitation, and teleassessment. The existing evidence on telehealth in ILD has focused primarily on remote monitoring strategies, such as home spirometry, wearable sensors, and oxygen saturation tracking, and, to a lesser extent, on telerehabilitation programs, with comparatively little attention given to teleassessment as a distinct clinical practice [5,10]. Teleassessment refers to the remote conduct of structured clinical evaluation, encompassing the assessment of functioning from a biopsychosocial perspective, patient-reported outcomes, and environmental and personal contextual factors, and is therefore conceptually distinct from both monitoring and rehabilitation [9]. Despite the growing literature on telemonitoring and telerehabilitation, little is known about the usability of structured teleassessment and the contextual factors that influence users’ experiences, particularly in individuals with ILD living in middle-income countries. To date, it remains unknown whether an ICF-based biopsychosocial teleassessment protocol delivered via video consultation is usable, acceptable, and contextually viable for individuals with ILD in Brazil, a middle-income country characterized by substantial socioeconomic and digital inequalities [3,6,7,8,9]. This knowledge gap has important clinical implications, as the implementation of teleassessment within hybrid models of care without understanding its usability and contextual determinants may inadvertently reinforce existing inequities in access to specialized ILD services and limit its effectiveness in routine practice [6,7,8,9,10,11,12].

Given these gaps, the present study aimed to investigate the association between contextual factors and teleassessment usability in individuals with ILD. Because usability is influenced not only by measurable perceptions but also by contextual experiences and individual perspectives, a mixed-methods approach was adopted to provide a more comprehensive understanding of this phenomenon. Additionally, it sought to integrate quantitative evidence, through TUQ-Brazil scores and contextual variables, with qualitative data derived from participant reports; to explore how socioeconomic, structural, and experiential factors influence the teleassessment experience. This integrated approach contributes to advancing the understanding of usability determinants in populations with complex chronic conditions and should be interpreted as an exploratory investigation in this field.

## 2. Materials and Methods

### 2.1. Study Design

This study adopted a convergent parallel mixed-methods design, in which quantitative and qualitative data were collected simultaneously, analyzed independently, and integrated during the interpretation phase. The approach combined a descriptive qualitative component, grounded in the constructivist–interpretivist epistemological paradigm, with quantitative analysis of TUQ-Brazil responses to evaluate teleassessment usability. A mixed-methods design was considered appropriate because usability is a multidimensional construct that encompasses both measurable outcomes and participants’ experiences, requiring the integration of quantitative indicators with qualitative insights to provide a comprehensive understanding of the acceptability and contextual viability of an ICF-based teleassessment protocol. The overall design and reporting followed the Good Reporting of A Mixed Methods Study (GRAMMS) recommendations [13].

### 2.2. Ethical Considerations

This study was approved by the Ethics Committee for Research Involving Human Subjects at Universidade Nove de Julho (CAAE: 83112624.9.0000.5511). All participants provided written informed consent electronically before participation. They were informed of their right to withdraw from the study at any time without penalty.

### 2.3. Setting

Data collection was conducted remotely through individual interviews via WhatsApp video calls between October 2024 and October 2025. Recruitment was carried out through broad dissemination of the study on social media, through informational materials targeted at individuals with chronic pulmonary disease.

### 2.4. Participants

Participants were eligible if they were adults (≥18 years), regardless of sex, demonstrated adequate cognitive capacity to participate in synchronous online interviews, and had access to home internet and a mobile device capable of supporting videoconferencing. The use of supplemental oxygen therapy was not considered an exclusion criterion. Participants diagnosed according to American Thoracic Society/European Respiratory Society guidelines were included. It is acknowledged that requiring internet access and a compatible device as inclusion criteria, and excluding individuals without these resources, constitutes an inherent selection bias that limits the scope of barrier analysis. Individuals with the most severe digital access constraints were systematically excluded from or unable to complete participation; consequently, findings on barriers and facilitators reflect the experiences of a digitally connected subgroup and likely overestimate acceptability relative to the broader ILD population. Physical limitations were not applied as exclusion criteria, and participants were permitted to receive technical assistance from family members during the session.

Participants were recruited using a combined purposive and convenience sampling strategy. Individuals meeting the predefined eligibility criteria were consecutively recruited among those available and willing to participate during the study period.

Interested individuals contacted the research team and underwent an initial screening to verify eligibility criteria. Eligible participants received detailed information about the study and, upon agreement and signing of the informed consent form, were included in the study.

### 2.5. Bias

Potential sources of information bias and social desirability bias were considered during study design and data collection. To minimize these effects, all interviews were conducted by the same trained researcher following a standardized study protocol. Participants were informed that there were no right or wrong answers and were encouraged to respond openly based on their own experiences. Standardized quantitative instruments and predefined interview procedures were used to promote consistency throughout data collection.

### 2.6. Sample Size

No formal sample size calculation was performed, as this study was designed as an exploratory, feasibility-oriented investigation. This approach is consistent with methodological recommendations for convergent mixed-methods studies and feasibility research in rare or low-prevalence clinical populations, where the primary objective is to explore usability, acceptability, and implementation issues rather than to test predefined hypotheses. The Final sample comprised 31 participants, a size comparable to that reported in previous telehealth feasibility and usability studies involving individuals with ILD and other chronic respiratory conditions, which have included samples ranging from 8 to 60 participants [14,15]. Findings should therefore be interpreted as exploratory and hypothesis-generating, and the observed associations should be confirmed in adequately powered future studies.

## 3. Variables

### 3.1. Dependent Variables

Usability was analyzed using the Brazilian version of the Telehealth Usability Questionnaire (TUQ-Brazil), a validated instrument designed to assess telehealth usability from both patient and healthcare professional perspectives [12]. The TUQ-Brazil consists of 21 items rated on a seven-point Likert scale, ranging from strong disagreement to strong agreement, and encompasses five key constructs: System Quality, User Interaction, User Satisfaction, Information Usability, and Perceived Usefulness. The overall usability score is calculated as the mean of all item responses, excluding those marked as not applicable. Higher mean scores approaching the upper limit of the seven-point scale indicate greater perceived usability of the evaluated telehealth system. The translation and cross-cultural adaptation followed standardized procedures, including forward translation, synthesis, back-translation, and expert committee review. The TUQ-Brazil has demonstrated adequate psychometric properties, supporting its validity and reliability for assessing telehealth usability in the Brazilian context [12].

### 3.2. Independent Variables

#### Teleassessment Variables

The ordinal scales used to characterize contextual variables were specifically developed by the authors for this study to support the mixed-methods data collection by operationalizing qualitative attributes into ordinal categories. These measures were intended for descriptive and exploratory purposes and were not subjected to pilot testing or formal psychometric validation. Variables addressing the logistical and structural factors of the remote format were operationalized as follows:

1. Resource Availability: Responses regarding the ownership of technological devices (smartphone, computer, laptop, tablet) were transformed into an ordinal scale from 0 to 4, where 0 indicates the absence of devices and 4 indicates possession of all of them.
2. Connectivity Cost: The monetary amounts reported by participants were categorized into cost ranges (BRL 0–50, BRL 51–100, BRL 101–200, and BRL 201 or more), forming an ordinal scale for financial impact analysis. For international comparability, these amounts correspond approximately to USD 0–10, USD 11–20, USD 21–40, and ≥USD 41, respectively.
3. Time spent traveling to an in-person appointment: The estimated travel time to an in-person assessment was converted into an ordinal scale from 1 to 4 (<15 min; 15 to 30 min; 31 to 60 min; and >60 min) to analyze the logistical impact.
4. Satisfaction and Feasibility: The willingness to use teleassessment again was converted into a scale from 1 to 4 (“Not willing” to “Very willing”). The perceived feasibility of teleassessment was analyzed as a dichotomous variable coded as 1 = No and 2 = Yes.
5. Accessibility for remote assessment and difficulty accessing technology: Internet access was scaled into three levels: 1 (no access), 2 (sometimes requests access), and 3 (has access). The difficulty generated by the lack of access was measured on an ordinal scale from 1 (“High impact”) to 3 (“No impact”).
6. Number of barriers to teleassessment access: The main barriers were summed into an ordinal score from 0 to 4, based on the number of obstacles faced. Intensity of barriers to teleassessment access was measured on a scale of 1 to 3, reflecting the impact of these barriers: 1 (“No impact”) to 3 (“High impact”).
7. Number of facilitators for teleassessment access: Facilitators were transformed into scores from 0 to 4, reflecting their presence. Intensity of facilitators to teleassessment access was classified on a scale from 1 (“No impact”) to 3 (“High impact”).
8. Influence of socioeconomic status: The influence of socioeconomic conditions was transformed into scores from 1 to 4 (“No influence” to “High influence”).

### 3.3. Data Sources and Measurement

Data were collected and analyzed by a single trained male researcher with postgraduate education and clinical experience in respiratory physiotherapy, who had no prior relationship with the participants. The interviewer had formal training in qualitative research methods, acquired through instruction in ATLAS.ti Desktop for Windows, version 25 (ATLAS.ti Scientific Software Development GmbH, Berlin, Germany) and an extension course in NVivo (versão 14, Lumivero), undertaken to ensure procedural consistency prior to data collection. Participants were informed about the study objectives and the researcher’s role before the interviews. In addition, prior training sessions were conducted between the interviewer and a research team member to ensure standardized and appropriate implementation of the study protocol.

After eligibility was confirmed, participants completed a secure questionnaire addressing sociodemographic and clinical characteristics. Individual remote interviews were scheduled within a maximum interval of two days and lasted from 40 to 80 min, depending on participant engagement. Each session began with the application of a biopsychosocial assessment protocol based on the ICF, incorporated as part of a broader assessment framework not analyzed in the present study. Furthermore, a semi-structured interview guide was followed, consisting of four open-ended questions: two focused on barriers and two on facilitators of the remote assessment. Subsequently, a questionnaire addressing factors related to telehealth was administered, including questions on the availability of technological resources, internet costs, time spent traveling for in-person appointments, satisfaction with telehealth services, accessibility to technology and internet access, as well as barriers and facilitators to access. The interviewer read the questions aloud and presented the response options, allowing participants to indicate their answers. Responses and observable behaviors were documented in real time through field notes, without audio or video recording, to preserve confidentiality. The interviewer had no prior contact with the participants, and an independent researcher conducted a validation procedure to strengthen the reliability of the study.

### 3.4. Data Analysis

Demographic and clinical characteristics were summarized using descriptive statistics in IBM SPSS Statistics (version 26). Categorical variables were presented as absolute frequencies and percentages, whereas continuous variables were described as means and standard deviations. Domain-level analysis involved examining the distribution of response categories across items within each usability domain, allowing the identification of patterns in participants’ perceptions while preserving the variability of individual responses. This descriptive approach facilitated the interpretation of usability across domains without imposing assumptions of parametric distribution on Likert-scale data.

#### 3.4.1. Quantitative Variables and Statistical Methods

The quantitative data derived from TUQ-Brazil were analyzed descriptively across all items. Each item, rated on a seven-point Likert scale, was categorized into three groups to facilitate interpretation: disagreement (scores 1–3), neutrality (score 4), and agreement (scores 5–7), as recommended for user experience analysis. Absolute frequencies were calculated for each category.

Divergent stacked bar charts were constructed to visually represent the distribution of responses across TUQ-Brazil items. Responses were displayed along a central zero reference line, with disagreement positioned to the left, neutral responses centered, and agreement positioned to the right. This approach allowed the identification of patterns of response concentration and dispersion across items, including usefulness, ease of use and learnability, interface quality, interaction quality, reliability, and satisfaction/future use, as well as variability across specific items.

The correlation between reported logistical conditions and the continuous TUQ-Brazil usability score was examined using Spearman’s rank correlation coefficient (rho), an appropriate nonparametric measure for assessing monotonic associations between ordinal variables and a continuous outcome. For the dichotomous logistical variable, Spearman’s rho is mathematically equivalent to a rank-biserial correlation. Correlation coefficients were interpreted as weak (<0.40), moderate (0.40–0.69), and strong (≥0.70), according to Dancey and Reidy [16].

#### 3.4.2. Missing Data

Missing data were assessed following recommendations for observational studies. For the Spearman correlation analyses, complete-case analysis was performed. Responses classified as “Not applicable” or “Don’t know” were treated as missing values and excluded only from the corresponding analysis. Consequently, the sample size varied slightly across correlation analyses according to data availability.

Regarding spirometric variables, 4 of the 31 participants (12.9%) had missing data due to the unavailability of pulmonary function reports. The missingness was considered to be at random (MAR), as it was related to the availability of previous examinations rather than participants’ respiratory outcomes. Spirometric variables were used exclusively for sample characterization; therefore, these missing data did not affect the inferential analyses. Descriptive analyses of spirometric parameters were performed using complete-case data from the 27 participants with available measurements.

#### 3.4.3. Qualitative Analysis

Data collection and analysis were conducted concurrently and guided by the principle of thematic saturation, defined a priori as the point at which no new codes or themes emerged in successive interviews [17]. Saturation was assessed through the concurrent analysis of interview transcripts, with each successive interview compared against the existing coding framework. Data collection ceased when no new codes or themes emerged in consecutive interviews. The final sample size was determined by the achievement of saturation, which occurred within approximately 91–100% of the planned interviews, indicating sufficient depth and completeness of the data. Participants’ responses and field notes were documented and transcribed in real time using Microsoft Word. To ensure confidentiality, all participants were anonymized and assigned unique identification codes (e.g., ID01, ID02). No audio or video recordings were performed to preserve privacy; therefore, data collection relied on real-time transcription complemented by detailed field notes to ensure accurate capture of participants’ narratives.

Thematic analysis was conducted using ATLAS.ti Desktop for Windows, version 25 (ATLAS.ti Scientific Software Development GmbH, Berlin, Germany), following a recursive and iterative process beginning with repeated readings to promote familiarity with the data. The AI-assisted tool NotebookLM was used exclusively for a preparatory, non-interpretive function: converting real-time transcription documents into plain-text format compatible with ATLAS.ti and supporting preliminary dataset organization. All coding, interpretation, and thematic construction were performed by the interviewer-researcher, who read the transcripts independently and generated initial codes through inductive analysis without reliance on AI-generated outputs. The AI tool did not perform coding, did not assign themes, and was not used to interpret data. Subsequently, all codes and emerging themes were reviewed and refined by an independent researcher to strengthen analytical rigor. Member checking was conducted during the interviews by summarizing participants’ responses and asking them to confirm that the researcher’s understanding accurately reflected their intended meaning. This procedure was undertaken with all participants before concluding each interview to enhance the credibility and accuracy of the qualitative data. An audit trail was maintained through systematic documentation of field notes, code revision records, and reflexive memos. To enhance trustworthiness, reflexivity was addressed by acknowledging the research team’s clinical experience in respiratory rehabilitation as a potential influence on data interpretation. Analytical rigor was reinforced through external validation, systematic documentation of field notes, and the inclusion of rich, descriptive excerpts with verbatim quotations. Findings were further validated through participant checking, ensuring consistency between the data and their interpretation. It is important to note that no audio or video recordings were made; data were captured exclusively through real-time transcription and detailed field notes, which represent an inherent limitation of the qualitative data collection procedure.

### 3.5. Integration of Quantitative and Qualitative Data

Quantitative and qualitative data were analyzed independently and subsequently integrated during interpretation using a convergent mixed-methods approach. Quantitative findings identified patterns of teleassessment usability and their associations with contextual variables, whereas qualitative findings were used to explain, contextualize, and expand these results by providing participants’ perspectives on the factors influencing their teleassessment experience. Integration occurred through comparison of convergence, complementarity, and divergence between both datasets, informing the overall interpretation of the findings [13].

## 4. Results

Thirty-one individuals diagnosed with ILD, defined according to the criteria of the American Thoracic Society and the European Respiratory Society, were included. Participants were from four of the five major Brazilian geographic regions (Southeast, South, Central-West, and Northeast); no participants from the northern region were enrolled despite recruitment efforts targeting all regions. The Southeast region was predominant, accounting for 61.3% of the sample, which limits the generalizability of findings to contexts with greater geographic isolation and lower digital infrastructure. Results should therefore be interpreted as predominantly reflecting the experiences of participants from the Southeast with preserved digital access, rather than as representative of the full national ILD population. Most participants were women who self-identified as White. The majority had completed high school. Household incomes ranged from two to three minimum wages. All participants owned a smartphone and had internet access. Table 1 summarizes the sociodemographic and clinical characteristics of the sample.

Values are described as mean ± standard deviation (SD) for variables with a parametric distribution, according to the Shapiro–Wilk test.

**Figure 1:**
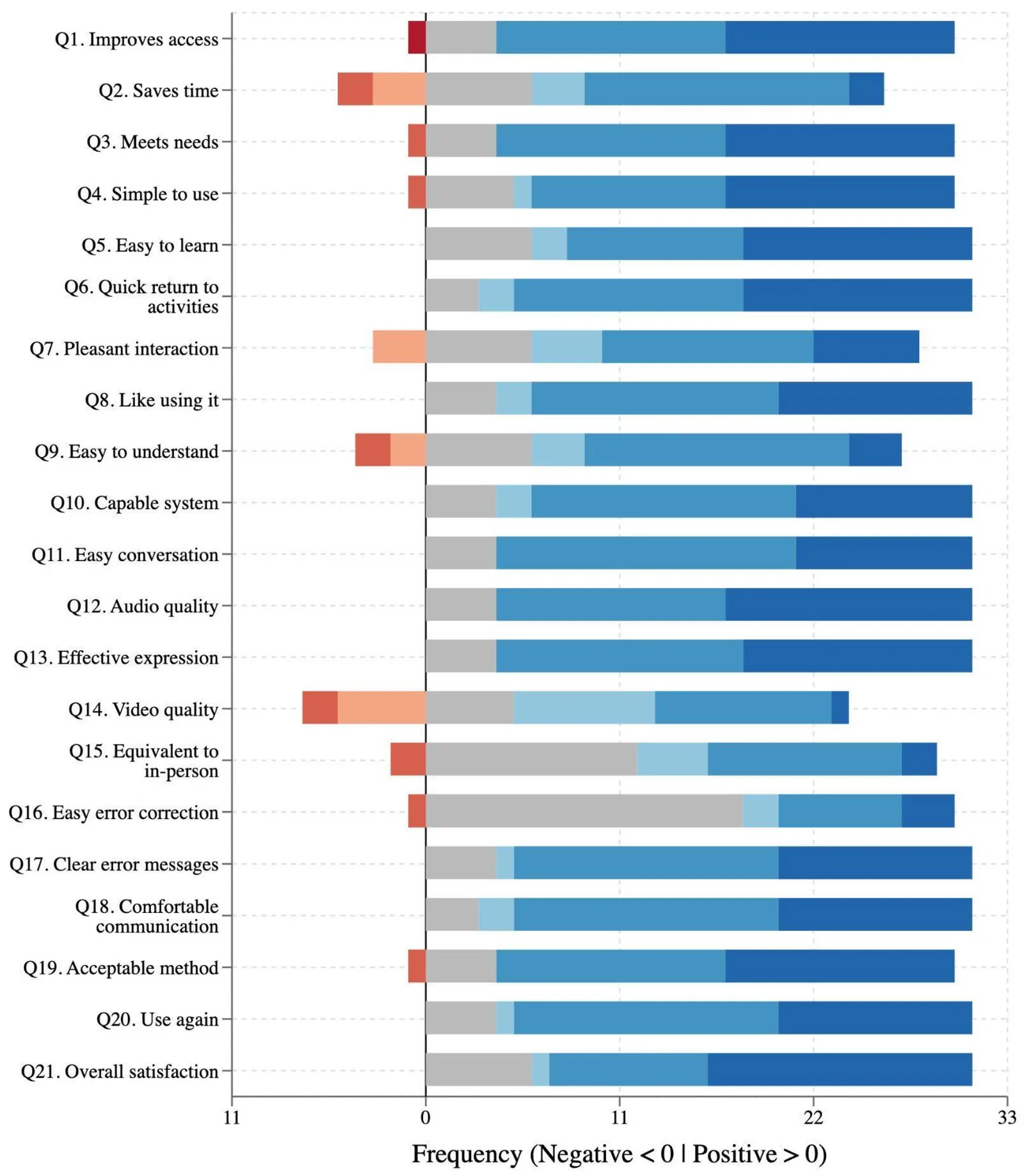
Diverging stacked bar chart of TUQ responses. Data from *n* = 31 participants. Each colored segment represents the frequency of responses for one Likert category. Red shades correspond to disagreement (scores 1 = strongly disagree, 2 = disagree, and 3 = somewhat disagree), gray represents neutral responses (score 4), and blue shades correspond to agreement (scores 5 = somewhat agree, 6 = agree, and 7 = strongly agree). Darker shades indicate stronger levels of disagreement or agreement. The three participants who selected the neutral option (score 4) for all 21 TUQ items are reflected in the gray segments across all bars.

The distribution of responses to the TUQ-Brazil showed a predominance of positive evaluations in all usability constructs, as described in Figure 1. Most items demonstrated predominantly positive responses, particularly those related to usefulness, ease of use, interaction quality, and satisfaction. Greater response variability was observed for video quality, pleasantness of interaction, ease of understanding, and perceived equivalence with in-person care.

Spearman correlation analysis identified a moderate positive association between income and TUQ-Brazil usability scores (rho = 0.437; *p* = 0.014), indicating that participants with higher income reported better perceived usability of teleassessment. To better understand this association, quantitative and qualitative findings were integrated in a joint display (Table 2).

### 4.1. Descriptive Analysis of Teleassessment Variables

Descriptive analysis indicated generally favorable conditions for teleassessment implementation (Table 3). Nearly all participants considered the format feasible (93.5%) and reported having access to the technological resources required for remote assessment. Although approximately one-third of participants identified at least one access barrier, most perceived its impact as low. Facilitators were reported by most participants, predominantly with low perceived intensity, suggesting that teleassessment was generally viewed as accessible within the study context despite the presence of individual contextual challenges.

### 4.2. Correlation Between Access Conditions and Usability (TUQ-Brazil)

The quantitative analyses revealed two exploratory associations between TUQ scores and contextual variables (*n* = 31). Greater perceived feasibility of the teleassessment format (rho = 0.397; *p* = 0.027), whereas greater perceived intensity of access barriers was associated with lower TUQ scores (rho = −0.428; *p* = 0.016). The remaining teleassessment-related variables showed no statistically significant associations with TUQ scores (*p* > 0.05) (Table 4).

### 4.3. Integration of Quantitative and Qualitative Findings

To explain the statistically significant quantitative associations, qualitative findings were integrated into a joint display. Representative verbatim quotations supporting each theme and subtheme are presented in Table 5. Higher perceived feasibility was consistently associated with participants’ perception that teleassessment was appropriate for follow-up care. Conversely, lower perceived feasibility was mainly attributed to the absence of physical examination, leading participants to view teleassessment as complementary rather than substitutive care. Likewise, greater perceived access barriers reflected technological difficulties, dependence on family support, emotional stress, unstable internet connectivity, and financial constraints. Together, these integrated findings indicate that teleassessment usability is shaped not only by platform characteristics but also by clinical expectations and broader contextual factors influencing engagement with remote care.

## 5. Discussion

This study investigated the usability of a biopsychosocial teleassessment protocol grounded in the ICF among individuals with ILD in Brazil, integrating quantitative usability scores with qualitative accounts of participants’ experiences. Rather than centering on the observation that participants generally reported favorable evaluations of teleassessment, the central contribution of this study lies in demonstrating that teleassessment usability was associated with contextual determinants, particularly income, perceived clinical feasibility, and the perceived intensity of access barriers. This finding reframes the interpretation of usability away from a purely technical or satisfaction-based construct and toward one that is embedded in social and structural conditions.

Although the divergent stacked-bar analysis showed a predominance of agreement across most TUQ-Brazil items, this pattern coexisted with neutral responses, dispersion in items related to video quality and equivalence to in-person care, and reports of meaningful limitations in the qualitative data. Categorical descriptions such as “high usability” would therefore overstate the homogeneity of participants’ experiences. The present findings are more accurately characterized as generally positive usability perceptions, with usability varying according to socioeconomic conditions and perceived clinical feasibility rather than being uniformly high across the sample.

A central finding of this study concerns the distinction between technological access and effective digital inclusion. All 31 participants owned a smartphone and reported having internet access, a level of technological availability that might suggest readiness for teleassessment. However, qualitative accounts revealed a markedly different picture: dependence on family members to operate devices or troubleshoot connectivity, operational difficulties among participants with limited digital skills, unstable connections requiring physical repositioning near a router, and stress or initial mistrust toward unfamiliar remote-care procedures. This contrast between formal access and effective use is consistent with the broader concept of the digital divide. Recent studies have shown that socioeconomic inequalities, digital literacy, and connectivity quality continue to create disparities in telehealth use and experience even among individuals who have internet access and compatible devices [18,19]. Our findings extend this perspective by demonstrating that these contextual factors influence not only access to telehealth but also the perceived usability of teleassessment itself. The implication for ILD care is that device ownership statistics, frequently used as proxies for digital readiness in telehealth planning, are insufficient to characterize a population’s actual capacity to engage effectively with remote assessment.

A second key finding relates to the nature of the limitation’s participants identified. Contrary to an assumption that usability barriers in telehealth are primarily technological, participants in this study most frequently emphasized a clinical limitation: the absence of physical examination. Statements referring to the impossibility of auscultation, palpation, or other hands-on procedures were central to participants’ accounts of when teleassessment was, or was not, considered adequate. This suggests that perceived usability in this population is not determined solely by platform quality or ease of use, but importantly by the perceived clinical adequacy of the remote modality for a condition as complex as ILD, which requires ongoing physical and respiratory monitoring. Importantly, this concern did not translate into outright rejection of teleassessment. Participants’ narratives instead reflected conditional acceptance: teleassessment was considered useful for follow-up, triage, and lower-complexity consultations, while in-person care remained the preferred option for situations requiring physical examination. This pattern aligns with calls in the broader telehealth literature for hybrid models of care that combine remote and in-person modalities according to clinical need, rather than treating these approaches as mutually exclusive alternatives [20,21].

The correlation analysis revealed an additional nuance: the intensity of perceived barriers was significantly associated with usability, whereas the number of barriers identified was not. This dissociation suggests that the perceived intensity of barriers may be more relevant than simply the number of reported barriers. A single barrier experienced as highly disruptive for instance, a connection failure during a clinically meaningful moment of the assessment, or a feeling of distrust toward the format may compromise the overall usability experience more substantially than several minor inconveniences that are easily resolved. This finding has practical implications for service design: efforts to improve teleassessment usability may benefit more from addressing the severity of specific, high-impact barriers than from attempting to eliminate every minor obstacle reported by users. Although exploratory, this finding suggests that users’ perceptions of how strongly these barriers affect their teleassessment experience may represent an important aspect to consider when implementing teleassessment in clinical practice. Future studies should investigate this relationship in larger and more diverse samples.

The association between income and usability reinforces the role of socioeconomic determinants identified throughout the qualitative data. However, this relationship should not be reduced to the capacity to purchase devices. Participants’ accounts suggest that income shapes teleassessment usability through several interconnected pathways: stability and quality of internet connectivity, the type of device available (a computer versus a smartphone screen), continuity of access across consecutive appointments, and, in several cases, whether teleconsultations were facilitated through private health insurance rather than out-of-pocket arrangements. These pathways suggest that socioeconomic disadvantage may operate cumulatively across multiple dimensions of the teleassessment experience, rather than through device ownership alone; given the cross-sectional design, this pathway should be interpreted as a plausible mechanism rather than an established causal sequence.

These findings carry relevance for the clinical profile of individuals with ILD. Dyspnea, fatigue, exercise intolerance, and progressive functional limitation frequently make travel to healthcare facilities physically demanding and logistically burdensome for this population [22]. In this context, the convenience afforded by teleassessment may carry clinical relevance beyond what would be expected in populations without significant mobility constraints, consistent with previous work highlighting the value of remote monitoring and telehealth strategies in ILD care [23]. However, this same population reported heightened sensitivity to the absence of physical examination given the respiratory nature of their condition.

Beyond the dimensions captured by standardized usability instruments, participants’ narratives revealed emotional and relational factors that conventional usability metrics may not fully capture: insecurity, stress associated with unfamiliar procedures, initial distrust toward the legitimacy of the remote-care request, and reliance on the support of family members to participate. These elements expand the understanding of what shapes the teleassessment experience in populations with chronic respiratory disease and suggest that future usability frameworks for this population may benefit from incorporating emotional and relational dimensions alongside conventional system-quality and interaction constructs.

These findings should be situated within the broader literature on telehealth in low- and middle-income countries. Much of this literature has emphasized technological availability as the principal determinant of telehealth adoption, with comparatively less attention to how social inequalities shape the practical, day-to-day experience of using these services once access has nominally been achieved [24]. The present findings suggest that, at least for teleassessment in ILD, the more clinically actionable question may not be whether patients have a smartphone and an internet connection, but whether they can use that connection reliably, autonomously, and without disproportionate burden relative to wealthier counterparts.

Taken together, these results caution against an overly optimistic narrative regarding telehealth expansion in chronic respiratory care. While teleassessment demonstrated meaningful potential in this sample, its appropriate implementation appears contingent on adequate infrastructure, accessible technical support, attention to digital literacy, and clear clinical criteria delineating when remote assessment is appropriate and when in-person evaluation remains necessary. Without these safeguards, expanding teleassessment risks widening, rather than narrowing, existing inequities in access to respiratory care.

This study has several limitations that should be considered when interpreting the findings. First, multiple exploratory correlation analyses were performed without adjustment for multiple comparisons. Given the exploratory nature of this study, adjustments for multiple comparisons were not applied because the primary objective was hypothesis generation rather than confirmatory hypothesis testing. Consequently, statistically significant associations should be interpreted with caution, recognizing the increased possibility of type I error, and should be confirmed in future studies with larger samples and adequate statistical power. Second, qualitative data were collected through real-time transcription and detailed field notes without audio or video recordings. Although this approach was adopted to preserve participants’ privacy and confidentiality, it may have limited the richness and completeness of the qualitative data by preventing subsequent verification of participants’ exact wording and reducing opportunities to revisit interview interactions during analysis. To enhance the credibility of the findings, real-time transcription was complemented by detailed field notes, an audit trail, independent review of codes and themes, and participant checking. Nevertheless, future studies incorporating audio- or video-recorded interviews may provide greater depth and further strengthen qualitative data credibility. Third, although participants were recruited from four Brazilian geographic regions, all owned a smartphone and had internet access, indicating a digitally connected sample. Thus, the findings primarily reflect the usability of teleassessment among individuals with preserved digital access and may underestimate barriers experienced by socially vulnerable populations or those living in areas with more limited technological infrastructure. Future studies should specifically include digitally underserved groups to better characterize the equity and accessibility of teleassessment implementation in low- and middle-income settings. Finally, the relatively high educational attainment observed in this sample may have favorably biased usability perceptions relative to populations with lower digital literacy. These limitations, considered together, suggest that the present findings likely represent a conservative, rather than worst-case, estimate of the barriers that a broader ILD population would encounter when using teleassessment.

## 6. Conclusions

This mixed-methods study found that teleassessment usability among individuals with ILD in Brazil was generally perceived positively. Exploratory analyses identified statistically significant associations between teleassessment usability and income, perceived clinical feasibility, and the perceived intensity of access barriers. Qualitative findings further indicated that the absence of physical examination was the main perceived clinical limitation of teleassessment, reinforcing its role as a complementary strategy rather than a replacement for face-to-face care. Overall, these findings suggest that teleassessment usability should be understood not merely as a technological attribute, but as a contextual phenomenon shaped by socioeconomic conditions, clinical context, and users’ experiences. Given the exploratory nature of these findings and the limited sample size, they should be interpreted as hypothesis-generating and confirmed in future studies with larger, adequately powered samples. The mixed-methods design strengthened this interpretation by integrating quantitative associations with participants’ perspectives, providing a more comprehensive understanding of the contextual factors influencing teleassessment usability.

## Figures and Tables

**Table 1:** Characteristics of study participants (*n* = 31).

Characteristic	Value
Age (years)	51.58 ± 12.72
Sex	
Male	15 (48.4)
Female	16 (51.6)
Ethnicity	
White	15 (48.4)
Black	5 (16.1)
Mixed-race	11 (35.5)
Education level	
Elementary school I	2 (6.5)
Elementary school II	2 (6.5)
High school	10 (32.3)
Incomplete higher education	4 (12.9)
Completed higher education	7 (22.6)
Postgraduate	6 (19.4)
Monthly household income	
Up to one minimum wage	4 (12.9)
Two to three minimum wages	14 (45.2)
Three to five minimum wages	6 (19.4)
More than five minimum wages	7 (22.6)
Region of residence	
South	4 (12.9)
Southeast	19 (61.3)
Central-West	4 (12.9)
Northeast	4 (12.9)
Interstitial lung disease subtype	
Idiopathic pulmonary fibrosis	11 (35.5)
ILD associated with connective tissue disease	10 (32.3)
Sarcoidosis	4 (12.9)
Interstitial pneumonia	3 (9.7)
Progressive pulmonary fibrosis	2 (6.5)
Hypersensitivity pneumonitis	1 (3.2)
Pulmonary function	
FVC (L)	2.51 ± 0.93
FVC (% predicted)	59.57 ± 19.45
FEV_1_ (L)	2.09 ± 0.75
FEV_1_ (% predicted)	62.19 ± 19.57
FEV_1_/FVC (% predicted)	103.45 ± 11.20
Oxygen therapy	
Yes	9 (29.0)
No	22 (71.0)
Oxygen flow (participants receiving oxygen therapy, *n* = 9)	
1 L/min	3 (33.3)
2–3 L/min	2 (22.2)
3–4 L/min	4 (44.4)
Technology access	
Owns a mobile phone	31 (100.0)
Internet access	31 (100.0)

**Table 2:** Joint Display of the Association between Income and Teleassessment Usability.

Quantitative Finding	Qualitative Evidence	Meta-Inference
Higher income was associated with greater teleassessment usability (rho = 0.437; *p* = 0.014; moderate correlation).	**Access to adequate devices:** “If I had a computer, it would help me see better than through my cell phone screen…” (ID9) **Internet access:** “For those who don’t have the internet, it can be difficult… those people would have difficulty.” (ID15) **Financial resources:** “Unless you have resources, but those are things that not everyone can have.” (ID16) “If you have the ‘money,’ we have better things, better service.” (ID25) **Health insurance and affordability:** “The financial aspect has a moderate influence because most of the teleconsultations I did were through the health insurance plan.” (ID27)	Economic resources influenced teleassessment usability by determining access to appropriate devices, stable internet connectivity, and private health services, thereby shaping participants’ ability to engage effectively with remote assessment.

**Note:** rho = Spearman correlation coefficient. TUQ-Brazil = Telehealth Usability Questionnaire (Brazilian Version). Values in bold indicate statistical significance (*p* < 0.05).

**Table 3:** Frequency Distribution of Teleassessment Variables.

Variable	Response	*n* (%)
Feasibility	Yes	29 (93.5)
	No	2 (6.5)
Resource availability	1 resource	7 (22.6)
	2 resources	13 (41.9)
	3 resources	7 (22.6)
	4 resources	4 (12.9)
Monthly connectivity costs	USD 0–10	1 (3.2)
	USD 10–20	8 (25.8)
	USD 20–40	16 (51.6)
	>USD 40	6 (19.4)
Travel time to an in-person appointment	<15 min	2 (6.5)
	15–30 min	7 (22.6)
	31–60 min	11 (35.5)
	>60 min	11 (35.5)
Willingness to switch to teleassessment	Not willing	2 (6.5)
	Slightly willing	2 (6.5)
	Willing	16 (51.6)
	Very willing	11 (35.5)
Accessibility for teleassessment	Has access	31 (100.0)
Difficulty accessing technology	No difficulty	27 (87.1)
	Low	3 (9.7)
	High	1 (3.2)
Number of barriers to teleassessment	None	21 (67.7)
	One	8 (25.8)
	Two	1 (3.2)
	Three	1 (3.2)
Barrier intensity	Not applicable	5 (16.1)
	No impact	18 (58.1)
	Low	6 (19.4)
	High	2 (6.5)
Influence of socioeconomic conditions	Don’t know	1 (3.2)
	No influence	8 (25.8)
	Little influence	6 (19.4)
	Moderate influence	7 (22.6)
	High influence	9 (29.0)
Number of facilitators for teleassessment	None	1 (3.2)
	One	5 (16.1)
	Two	9 (29.0)
	Three	14 (45.2)
	Four	2 (6.5)
Facilitator intensity	Not applicable	1 (3.2)
	Low	23 (74.2)
	Moderate	6 (19.4)
	High	1 (3.2)

**Table 4:** Spearman Correlation Analysis between Reported Teleassessment Logistical Conditions and TUQ-Brazil Usability Scores.

Variable	Spearman Coefficient (rho)	*p*-Value
Resource Availability	0.025	0.892
Connectivity Costs	0.227	0.220
Travel Time to In-Person Appointment	0.072	0.698
Willingness to Switch to Teleassessment	0.083	0.657
Feasibility	0.397 *	**0.027**
Difficulty Accessing Technology	0.121	0.517
Number of Barriers to Teleassessment Access	−0.197	0.289
Intensity of Barriers to Teleassessment Access	−0.428 *	**0.016**
Influence of Socioeconomic Condition	0.299	0.102
Number of Facilitators for Teleassessment Access	0.315	0.085
Intensity of Facilitators for Teleassessment Access	0.310	0.090

**Note:** TUQ = Telehealth Usability Questionnaire. The coefficient assesses rank covariance. * *p* < 0.05. Complete-case analysis was used for each correlation. Sample size varied slightly according to data availability.

**Table 5:** Joint Display Integrating Quantitative and Qualitative Findings on Teleassessment Usability.

Quantitative Finding	Qualitative Theme	Representative Quotations	Meta-Inference
**Perceived feasibility was positively associated with usability (rho = 0.397; *p* = 0.027).**	Teleassessment as complementary rather than substitutive care	“It does not address everything because it requires physical assessment.” (ID4) “Telehealth solves part of it but not everything.” (ID7) “Virtual care does not replace in-person care because of physical contact.” (ID20)	Participants accepted teleassessment as a complementary modality for follow-up but considered physical examination indispensable for comprehensive clinical evaluation.
**Greater perceived intensity of access barriers was associated with lower usability (rho = −0.428; *p* = 0.016).**	Technological difficulties and digital support	“It became a barrier because I did not know how to use it.” (ID3) “If I had been alone, I would not have been able to solve it.” (ID15)	Limited digital literacy and dependence on family support reduced participants’ autonomy and confidence during teleassessment.
	Emotional impact and stress	“At first I felt distrust, thinking it could be a scam.” (ID9) “It caused me worry and was stressful until I managed.” (ID8)	Emotional insecurity and stress emerged as experiential barriers that negatively influenced perceived usability despite successful completion of the assessment.
	Infrastructure and socioeconomic constraints	“If I did not have the financial means, I would not even have access.” (ID11) “Lack of digital literacy makes access more difficult.” (ID1)	Structural factors, including financial resources, internet access, and digital literacy, shaped teleassessment usability beyond the characteristics of the platform itself.

**Note:** rho = Spearman correlation coefficient. TUQ-Brazil = Telehealth Usability Questionnaire (Brazilian Version). Values in bold indicate statistical significance (*p* < 0.05).

## Data Availability

The data presented in this study are available on request from the corresponding author due to privacy and ethical restrictions.
